# Are Hot Drinks Cooling?

**Published:** 1875-10

**Authors:** 


Are Hot Drinks Cooling?—A Vicksburg boy got hold of a newspaper the other day, which said that hot drinks were more cooling to the system than cool beverages. He emptied a handful of ground pepper into the coffe-pot, in order to test the experiment, and soon after breakfast was heard confessing to his father his disbelief in domestic recipes of any kind whatever. The father used a barrel stave to aid his side of the argument.—*Pharmacal Gazette.*

